# Pharmacokinetics and Monte Carlo Simulation of Meropenem in Critically Ill Adult Patients Receiving Extracorporeal Membrane Oxygenation

**DOI:** 10.3389/fphar.2021.768912

**Published:** 2021-11-01

**Authors:** Jae Ha Lee, Dong-Hwan Lee, Jin Soo Kim, Won-Beom Jung, Woon Heo, Yong Kyun Kim, Se Hun Kim, Tae-Hoon No, Kyeong Min Jo, Junghae Ko, Ho Young Lee, Kyung Ran Jun, Hye Sook Choi, Ji Hoon Jang, Hang-Jea Jang

**Affiliations:** ^1^ Division of Pulmonology and Critical Care Medicine, Department of Internal Medicine, Inje University Haeundae Paik Hospital, Inje University College of Medicine, Busan, South Korea; ^2^ Department of Clinical Pharmacology, Hallym University Sacred Heart Hospital, Hallym University College of Medicine, Anyang, South Korea; ^3^ Division of General Surgery, Inje University Haeundae Paik Hospital, Inje University College of Medicine, Busan, South Korea; ^4^ Division of Cardiac Surgery, Inje University Haeundae Paik Hospital, Busan, South Korea; ^5^ Division of Infectious Diseases, Department of Internal Medicine, Hallym University Sacred Heart Hospital, Hallym University College of Medicine, Anyang, South Korea; ^6^ Department of Anesthesiology, Inje University Haeundae Paik Hospital, Inje University College of Medicine, Busan, South Korea; ^7^ Department of Infectious Diseases, Inje University Haeundae Paik Hospital, Inje University College of Medicine, Busan, South Korea; ^8^ Department of Endocrinology, Inje University Haeundae Paik Hospital, Inje University College of Medicine, Busan, South Korea; ^9^ Department of Pulmonology, Inje University Busan Paik Hospital, Inje University College of Medicine, Busan, South Korea; ^10^ Department of Laboratory Medicine, Inje University Haeundea Paik Hospital, Inje University College of Medicine, Busan, South Korea; ^11^ Division of Pulmonary, Allergy and Critical Care Medicine, Department of Internal Medicine, Kyung Hee University Medical Center, Seoul, South Korea

**Keywords:** meropenem, extracorporeal membrane oxygenation, population pharmacokinetics, Monte Carlo simulation, fT>MIC

## Abstract

**Objectives:** There have been few clinical studies of ECMO-related alterations of the PK of meropenem and conflicting results were reported. This study investigated the pharmacokinetics (PK) of meropenem in critically ill adult patients receiving extracorporeal membrane oxygenation (ECMO) and used Monte Carlo simulations to determine appropriate dosage regimens.

**Methods:** After a single 0.5 or 1 g dose of meropenem, 7 blood samples were drawn. A population PK model was developed using nonlinear mixed-effects modeling. The probability of target attainment was evaluated using Monte Carlo simulation. The following treatment targets were evaluated: the cumulative percentage of time during which the free drug concentration exceeds the minimum inhibitory concentration of at least 40% (40% fT_>MIC_), 100% fT_>MIC_, and 100% fT_>4xMIC_.

**Results:** Meropenem PK were adequately described by a two-compartment model, in which creatinine clearance and ECMO flow rate were significant covariates of total clearance and central volume of distribution, respectively. The Monte Carlo simulation predicted appropriate meropenem dosage regimens. For a patient with a creatinine clearance of 50–130 ml/min, standard regimen of 1 g q8h by i. v. infusion over 0.5 h was optimal when a MIC was 4 mg/L and a target was 40% fT_>MIC_. However, the standard regimen did not attain more aggressive target of 100% fT_>MIC_ or 100% fT_>4xMIC_.

**Conclusion:** The population PK model of meropenem for patients on ECMO was successfully developed with a two-compartment model. ECMO patients exhibit similar PK with patients without ECMO. If more aggressive targets than 40% fT_>MIC_ are adopted, dose increase may be needed.

## Introduction

Extracorporeal membrane oxygenation (ECMO) therapy provides life support to patients with cardiac, respiratory, or cardiopulmonary failure by adding oxygen and removing carbon dioxide (Squiers et al., 2016). The use of ECMO became important for critically ill patients during the 2009 H1N1 influenza pandemic and has increased remarkably since influenza pandemic by H1N1 virus in 2009 (Sauer et al., 2015; Raman and Dalton, 2016; Thiagarajan et al., 2017). However, ECMO use can put patients at increased risk of nosocomial infection as a result of cannulation of the major peripheral or central vessels to enable cardiopulmonary bypass (Bizzarro et al., 2011; Biffi et al., 2017). Therefore, many antibiotics are used in patients undergoing ECMO for prophylaxis or treatment. As the use of antibiotics in patients with ECMO increases, many studies have reported changes in the pharmacokinetic (PK) parameters of these agents, such as increased volume of distribution or altered clearance (Sherwin et al., 2016; Abdul-Aziz and Roberts, 2020).

There have been still few population PK studies on ECMO-related PK alterations of meropenem (Shekar et al., 2014; Hanberg et al., 2018; Gijsen et al., 2021). Meropenem, one of the antimicrobials typically used in ECMO patients, is a parenteral carbapenem antimicrobial agent and has a broad spectrum of antibacterial activity against Gram-positive, Gram-negative, and anaerobic pathogens. It is indicated for the treatment of complicated intra-abdominal infection, complicated skin and skin structure infections, bacterial meningitis, pneumonia, intra- and post-partum infections, and febrile neutropenia (Leroy et al., 1992; Wiseman et al., 1995; Hurst and Lamb, 2000; Lowe and Lamb, 2000; Baldwin et al., 2008; Mohr, 2008). The previous population PK studies did not find the effect of ECMO on meropenem PK, while in an *ex vivo* study, meropenem was degraded and significantly sequestered within the ECMO circuit after 4–6 h of treatment, and only 20% was recovered from the circuit at 24 h, compared to 40% of the control (Shekar et al., 2012). However, the significant effect might not have been found because the number of ECMO patients was too small, from 10 to 14, in those clinical studies. These indicate a lack of understanding of the PK changes and the appropriate dosing strategy for meropenem in patients undergoing ECMO therapy.

The aim of the present study was to develop a population PK model for meropenem and to evaluate pharmacodynamic (PD) target attainment in adults on ECMO by means of Monte Carlo simulations.

## Materials and Methods

### Patients

This was a prospective study conducted at the Department of Pulmonology and Clinical Care Medicine, Haeundae Paik Hospital, Busan, Republic of Korea, from November 2018 to November 2020. Thirty patients (aged ≥19) who underwent ECMO for respiratory and/or cardiac dysfunction and who received meropenem for treatment or prophylaxis were included in the clinical study. A written informed consent form was obtained from and signed by the legally authorized representative of each subject before enrollment. This study was approved by the Institutional Review Board of Inje University Haeundae Paik Hospital (IRB No. 2018-06-017) and conducted in accordance with the Declaration of Helsinki and Good Clinical Practice. The baseline demographic factors between continuous renal replacement therapy (CRRT) group and non-CRRT groups were compared. If the parameters for both the groups are normally distributed, the t-test was used; if either of the two groups did not satisfy the normality, the Wilcoxon rank-sum test was used.

### ECMO Apparatus

The ECMO system was the Permanent Life Support System (MAQUET, Rastatt, Germany) consisting of a PLS-i oxygenator and a ROTAFLOW Centrifugal Pump. The circuit was primed with 1 L of normal saline or plasma solution. The total circuit volume was 500–600 ml.

### Study Design

A single 500 or 1,000 mg dose of meropenem diluted in 200 ml of 5% dextrose in water was infused intravenously over 3 h to patients on ECMO. After the first dose. 7 blood samples were drawn from each patient’s arterial catheter into heparinized tubes. The predetermined three sampling schemes were as follows: Scheme 1 = 0 (predose), 3.33, 3.67, 4, 5, 6, and 8 h; Scheme 2 = 0, 3.33, 3.67, 4, 5, 8, and 10 h; or Scheme 3 = 0, 3.33, 3.67, 4, 6, 11, and 14 h after the start of meropenem administration. The blood sampling times were determined by considering the blood sampling times and distribution half-life (0.498 and 0.504 h) and elimination half-life (1.67 and 2.09 h) of two previous studies (Doh et al., 2010; Lu et al., 2016). The plasma samples were separated via centrifugation (2000g at 4°C for 10 min) within 30 min of sampling. They were transferred to polypropylene tubes by 1 ml and stored at −70°C for 1–6 months until assayed.

### Meropenem Assay

Plasma meropenem concentrations were analyzed using a validated high-performance liquid chromatography (HPLC)–tandem mass spectrometry assay. The HPLC was performed on an Agilent 1,200 series with an Atlantis C_18_ column (Company, Waters, Milford, MA, USA) (2.0 *mm* × 150 *mm*, 3.0 *μ*). Mass spectrometric detection was performed using a triple-quadrupole mass spectrometer (SCIEX API4000, Applied Biosystems, Foster City, CA) with an electrospray ionization interface. Data acquisition and processing were accomplished using Analyst software (version 1.4.1; Applied Biosystems, Foster City, CA). The lower limit of quantitation was 1 mg/L. The assay results were linear over a range of 1–50 mg/L (R^2^ = 0.9974). Inter-day precision and accuracy of the validation concentration range (1, 2, 5, 10, 25, and 50 mg/L) analyzed with standard samples for 3 days were 0.5–2.7% and 89.9–100.0%, respectively.

### Population PK Analysis

Population PK modeling was implemented using NONMEM 7.5 (Icon Development Solutions). A first-order conditional estimation with interaction method was used during analysis to account for potential interactions involving between-subject variability (BSV) for PK parameters and residual variability (RV), caused by assay error, model misspecification, errors in independent variables, and intra-individual variability, etc. One-, two-, and three-compartment models were tested using ADVAN1 TRANS2, ADVAN3 TRANS4, and ADVAN11 TRANS4 from the pharmacokinetic model library in NONMEM. First-order kinetics was assumed for all PK processes other than the zero-order infusion.

The PK parameters were assumed to follow a log-normal distribution. The parameter model was defined as θ_i_ = θ × exp(η_i_), where θ is the median value of the PK parameter, θ_i_ is an individual parameter, and η_i_ is a random effect that is assumed to be normally distributed with a mean of 0 and variance of ω^2^. Additive, proportional, and combined additive and proportional error models were tested for RV, which was assumed to be normally distributed with a mean of 0 and variance of σ^2^ (Dosne et al., 2016).

Model evaluation and selection were based on objective function values (OFVs) by NONMEM, relative standard errors for parameter estimates, shrinkage of BSV, and diagnostic goodness-of-fit plots. In a log-likelihood ratio test, an OFV reduction (ΔOFV) greater than 3.84 between two nested models with one degree of freedom or greater than 5.99 with two degrees of freedom was considered a significant model improvement. Diagnostic plots included conditional weighted residuals (CWRES) v. time, CWRES v. model-predicted population concentration (PRED), observation v. PRED, and observation v. model-predicted individual concentration (IPRED) (Hooker et al., 2007).

Perl-speaks-NONMEM software (version 5.0.0, available at https://uupharmacometrics.github.io/PsN/) was used to search for significant covariates and evaluate the final model using a visual predictive check and nonparametric bootstrap method. Stepwise forward selection and backward elimination were conducted to identify significant covariates for PK parameters. The statistical significance criteria were *p* < 0.01 (ΔOFV < -6.63 with 1 degree of freedom) for selection and *p* < 0.001 (ΔOFV >10.8 with 1 degree of freedom) for elimination. A significant covariate was required to have clinical relevance and to meet the statistical criteria. The tested covariates for all PK parameters were sex, age, weight, height, body surface area (BSA), serum protein level, serum albumin level, primary diagnosis, comorbidity, Acute Physiology and Chronic Health Evaluation II (APACHE II) score, Sequential Organ Failure Assessment Score (SOFA) score, presence of CRRT, ECMO flow rate, and ECMO type (veno-arterial or veno-venous). Serum creatinine level and renal function as estimated by applying the Cockcroft-Gault (CG), Chronic Kidney Disease Epidemiology Collaboration (CKD-EPI), modified CKD-EPI, Modification of Diet in Renal Disease (MDRD), and modified MDRD equations were tested only for total clearance. The modified CKD-EPI and MDRD values were calculated using individual body surface area (BSA) values, where BSA was calculated by applying the Du Bois formula. The final PK parameter estimates between continuous renal replacement therapy (CRRT) group and non-CRRT groups were compared. If the values for both the groups are normally distributed, the t-test was used; if either of the two groups did not satisfy the normality, the Wilcoxon rank-sum test was used.

Prediction- and variability-corrected visual predictive checks were performed by comparing the observed plasma concentrations with 80% prediction intervals from 1,000 simulated datasets applying the final PK model. Virtual observations were compared with prediction- and variability-corrected observed concentrations of meropenem to evaluate the model performance (Bergstrand et al., 2011). The nonparametric bootstrap method was used to evaluate the stability of the final model. The median and 95% confidence intervals for the parameter estimates of bootstrap samples (*n* = 2000) were generated to compare with the final PK parameter estimates. The R statistical software package (version 4.0.3, available at https://www.r-project.org/) was used for visualization and postprocessing of modeling output.

### PD Target Attainment

Two Monte Carlo simulations were conducted. The first simulation was implemented to evaluate the appropriateness of the recommended dosing regimen (for a creatinine clearance [CL_CR_] > 50 ml/min, 1 g q8h; for a CL_CR_ of 26–50 ml/min, 1 g q12 h; for a CL_CR_ of 10–25 ml/min, 500 mg q12 h; for a CL_CR_ < 10 ml/min, 500 mg q24 h) when treating adult patients infected with intra-abdominal infection by *Pseudomonas aeruginosa* (*P. aeruginosa*). A total of 10,000 individual PK parameters of virtual patients was generated assuming a log-normal distribution for each PK parameter or each covariate. Then, ten thousand MICs were randomly assigned to the virtual patients. The clinical breakpoint distribution of MICs set by the European Committee on Antimicrobial Susceptibility Testing (EUCAST) was used to simulate the MICs. The steady-state concentration-time profiles (in minutes) for the virtual patients were generated to investigate the probability of target attainment (PTA). The target index for meropenem is the cumulative percentage of a 24 h period during which the free (*f*) drug concentration exceeds the minimum inhibitory concentration (MIC) at steady-state conditions (*f*T_>MIC_) (Drusano, 2004). The tested targets were 40% *f*T_>MIC_, 100% *f*T_>MIC_, and 100% *f*T_>4xMIC_ for meropenem. A dosing regimen was considered optimal if the PTA is equal to or greater than 90%. The *f* was fixed at 0.98 (Ulldemolins et al., 2011).

The second simulation was implemented to investigate the optimal dosing regimen for the three targets. Thousand individual PK parameters were generated assuming a log-normal distribution for each PK parameter. The one of the finally selected two covariates, CL_CR_, was generated by applying a uniform distribution within the range 0–170 ml/min and the virtual patients were assigned to the six renal function groups (0–10, 10–25, 25–50, 50–90, 90–130, or 130–170 ml/min). The other covariate, ECMO flow rate, was fixed to the median value of 3.7 L/min. Then, the PTA for the generated steady-state concentration-time profiles were evaluated with various combinations of the three doses (0.5, 1, and 2 g), two infusion times (0.5 and 3 h), two dosing intervals (8 and 12 h), and MICs (0.060, 0.125, 0.25, 0.5, 1, 2, 4, 8, and 16 mg/L). The thousand parameters were also used to investigate the effect of ECMO flow rate on the PTA of 40% *f*T_>MIC_. The steady-state concentration-time profiles were generated at the flow rate of 2, 4, and 6 L/min and evaluated with various combinations of the three doses (0.5, 1, and 2 g), two dosing intervals (8 and 12 h), and MICs (0.060, 0.125, 0.25, 0.5, 1, 2, 4, 8, and 16 mg/L), while the infusion time was fixed to 0.5 h.

## Results

### Baseline Characteristics

Prospectively, 30 patients were enrolled in this study, of whom 10 patients received CRRT (Table 1). The most common primary diagnosis was pneumonia in both the non-CRRT group (*n* = 11) and the CRRT group (*n* = 5). Of the types of ECMO employed, venovenous (VV)-ECMO was used in 75% (*n* = 15) and venoarterial (VA)-ECMO in 25% (*n* = 5) in non-CRRT group and VV-ECMO used in 80% (*n* = 8) and VA-ECMO used in 20% (*n* = 2) in the CRRT group. Serum creatinine level and eGFR by MDRD, modified MDRD, CKD-EPI, and modified CKD-EPI of the patients with CRRT were significantly different from those of the patients without CRRT, while CLCR by Cockcroft-Gault formula was not. The median (IQR) ECMO flow rates of the non-CRRT and CRRT group were 3.62 (2.62-4.08) L/min and 3.83 (3.47-4.17), respectively, and were not significantly different (*p* = 0.6511). All patients received 1,000 mg of meropenem with the exception of one in the non-CRRT group, who received 500 mg.

**TABLE 1:** Patient characteristics (median(IQR))^a^.

Parameter	Non CRRT (*n* = 20)	CRRT (*n* = 10)	*p*-value
Sex	male 13/female 7	male 4/female 6	
Age (yr)	63.0 (54.0–78.5)	63.0 (55.3–65.8)	0.6125^d^
Height (cm)	165 (162–172)	162 (155–174)	0.4535^c^
Weight (kg)	67.9 (56.8–77.5)	66.5 (60.3–86.3)	0.5083^c^
Body surface area (m^2^)	1.74 (1.62–1.83)	1.70 (1.63–1.96)	0.7771^c^
Primary disease (n)			
Pneumonia	11	5	
Ventricular fibrillation	2		
Interstitial lung disease	1		
Pulmonary thromboembolism	1	1	
Aortic dissection	1	1	
Cholecystitis	1		
Hemopneumothorax	1		
Inhalation injury	1		
Sigmoid colon cancer	1		
Cellulitis		1	
Scrub typhus		1	
Hemoperitoneum		1	
ICU duration (days)	24.5 (13.5–31.5)	29.5 (16.5–58.3)	0.1498^c^
MV duration	23.5 (8.50–31.3)	25.0 (15.0–33.8)	0.2175^c^
SOFA score	10.0 (7.75–12.3)	12.0 (10.3–13.0)	0.0474^c^
APACHE II score	26.0 (23.0–28.3)	26.0 (23.5–29.8)	0.5472^c^
CRP (mg/dl)	8.58 (4.13–18.3)	12.9 (6.61–20.2)	0.7509^c^
Albumin (g/dl)	2.75 (2.50–3.10)	2.65 (2.33–2.80)	0.3113^c^
Scr (mg/dl)	1.03 (0.71–1.67)	1.83 (1.44–2.32)	0.0209^d^
CL_CR_, Cockcroft-Gault (ml/min)	69.6 (42.2–115)	43.4 (31.8–51.0)	0.0862^d^
GFR, MDRD (ml/min/1.73m^2^)	71.8 (38.1–106)	31.0 (28.5–37.3)	0.0064^d^
GFR, modified MDRD (ml/min)^b^	73.4 (42.1–99.7)	32.7 (30.4–37.5)	0.0010^c^
GFR, CKD-EPI (ml/min/1.73m^2^)	72.9 (38.4–112)	32.5 (29.5–42.8)	0.0094^c^
GFR, modified CKD-EPI (ml/min)^b^	77.8 (39.4–102)	33.6 (31.2–43.8)	0.0011^d^
ECMO type	VA 5/VV 15	VA 2/VV 8	
ECO duration (days)	15.5 (7.40–20.3)	9.00 (6.00–13.8)	0.0618^c^
ECMO flow rate (L/min)	3.62 (2.62–4.08)	3.83 (3.47–4.17)	0.6511^c^
ECMO revolutions per min	2,868 (2,490–3,038)	2,685 (2,368–2,963)	0.5091^d^

a Abbreviations: IQR, interquartile range; ICU, intensive care unit; MV, mechanical ventilation; CRRT, continuous renal replacement therapy; ECMO, extracorporeal membrane oxygenation; FiO_2_, fraction of inspired oxygen; aPTT, activated partial thromboplastin time; MDRD, Modification of Diet in Renal Disease; GFR, glomerular filtration; eGFR, estimated glomerular filtration rate; RPM, rotations per minute.

b The modified MDRD and CKD-EPI equations adjusted to individual BSA are GFR (ml/min) = GFR (MDRD or CKD-EPI) × (BSA/1.73 m^2^).

c independent t-test.

d Wilcoxon rank-sum test.

### Clinical Outcomes

The median (IQR) durations of ECMO therapy of the non-CRRT and CRRT groups were 15.5 (7.40–20.3) days and 9.00 (6.00–13.8) days, respectively. Ten patients (50%) in the non-CRRT group and five patients (50%) in the CRRT group died in the intensive care unit (ICU). The mortality rates for the patients in the non-CRRT group receiving VV-ECMO or VA-ECMO were 40% (*n* = 6) and 80% (*n* = 4), respectively. Those for CRRT group were 50% (*n* = 4) and 50% (*n* = 1), respectively. The most common causes of death were multi-organ failure and sepsis.

### Population PK Analysis

A total of 210 plasma samples was used to build the population PK model. The time course of meropenem concentrations was well described by a two-compartment model. The objective function values (OFVs) for one-, two-, and three-compartment models without covariates were 547.667, 409.786, and 395.043, respectively. However, the three-compartment model failed to achieve model convergence and generated poor parameter estimates. The structural PK parameters for the two-compartment model were total clearance (CL), central volume of distribution (V_C_), volume of distribution for the peripheral compartment (V_P_), and intercompartmental clearance between V_C_ and V_P_ (Q), as shown in Table 2. All PK parameter estimates were not significantly different between the CRRT and non-CRRT groups (Table 3). Individual model fits are shown in Supplementary Figure S1.

**TABLE 2:** Population PK parameter estimates for meropenem in ECMO patients^a^.

Parameter	Estimates	RSE (%)	Bootstrap median (95% CI)
Structural model			
CL = θ_1_ × (1 + θ_2_ × (CG - 49.7))			
θ_1_ (L/h)	7.35	7.33	7.28 (6.38–8.40)
θ_2_	0.0104	30.3	0.0107 (0.00582–0.0182)
V_C_ = θ_3_ × (1 + θ_4_ × (LPM - 3.7))			
θ_3_ (L)	17.3	10.9	17.2 (13.9–21.7)
θ_4_	0.337	7.10	0.334 (0.224–0.383)
Q (L/h)	14.5	10.8	14.5 (10.5–19.3)
V_P_ (L)	12.8	9.32	12.8 (10.3–15.6)
Between-subject variability			
ω_CL_ (%)	39.6	17.4	36.5 (22.1–50.4)
ω_Vc_ (%)	48.5	17.1	46.5 (30.6–63.5)
ω_Vp_ (%)	38.8	18.2	37.7 (23.8–52.2)
Residual variability			
σ_Additive error_ (mg/L)	0.370	30.3	0.356 (0.000–0.619)
σ_Proportional error_ (%)	4.73	7.10	4.73 (2.85–6.27)

a Abbreviations: ECMO, extracorporeal membrane oxygenation; RSE, relative standard error; CL, total clearance; V_C_, central volume of distribution; V_P_, peripheral volume of distribution; Q, intercompartmental clearance between V_C_ and V_P_; CG. glomerular filtration rate estimated by Cockcroft-Gault equation; LPM (L/min), ECMO flow rate (L/min).

**TABLE 3:** Comparison of the final PK parameter estimates between non-CRRT and CRRT groups^a^.

Parameter	Non CRRT	CRRT	*p*-value
CL	8.57 (6.56–13.4)	6.03 (5.56–7.18)	0.0585^c^
V_C_ (L)	13.3 (9.24–16.3)	21.7 (12.5–29.7)	0.2349^c^
V_P_ (L)	12.6 (11.2–15.3)	13.3 (12.0–17.0)	0.4202^b^
V_SS_ (L)	25.0 (20.4–32.3)	35.4 (29.6–42.4)	0.0529^c^

a Abbreviations: CRRT, continuous renal replacement therapy; CL, total clearance; V_C_, central volume of distribution; V_P_, peripheral volume of distribution; Q, intercompartmental clearance between V_C_ and V_P_.

b independent t-test.

c Wilcoxon rank-sum test.

In the final PK model (OFV = 369.100), CL_CR_ as estimated by the Cockcroft-Gault formula was identified as a significant covariate for CL, while the OFV of a reduced model without this covariate increased to 391.854. The random BSV for CL was reduced from 57.9 to 39.6% after including the covariate. The ECMO flow rate was a significant covariate for V_C_, while the OFV of a reduced model without ECMO flow rate on V_C_ increased to 387.207. The random BSV for V_C_ was reduced from 67.1 to 48.5% after including the covariate. The RV was well described by a combined additive and proportional error model. Model robustness was supported by the bootstrap median values and the 95% confidence intervals for the parameter estimates (Table 2).

**FIGURE 1:**
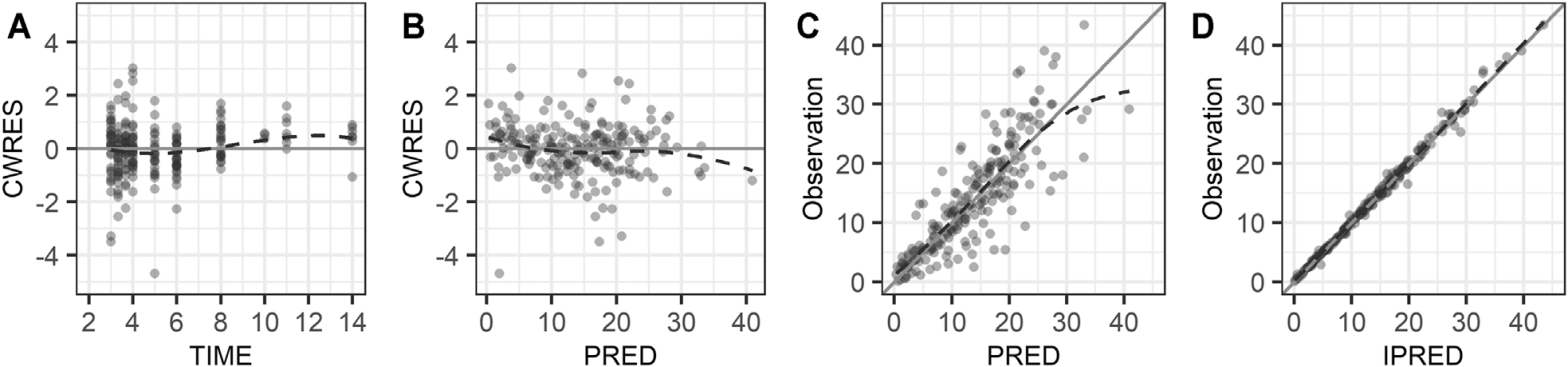
Goodness of fit plots: **(A)** conditional weighted residuals versus time, **(B)** conditional weighted residuals versus population predicted concentration, **(C)** observed concentration versus population predicted concentration, and **(D)** observed concentration versus individual predicted concentration. The dashed lines are smooth curves.

**FIGURE 2:**
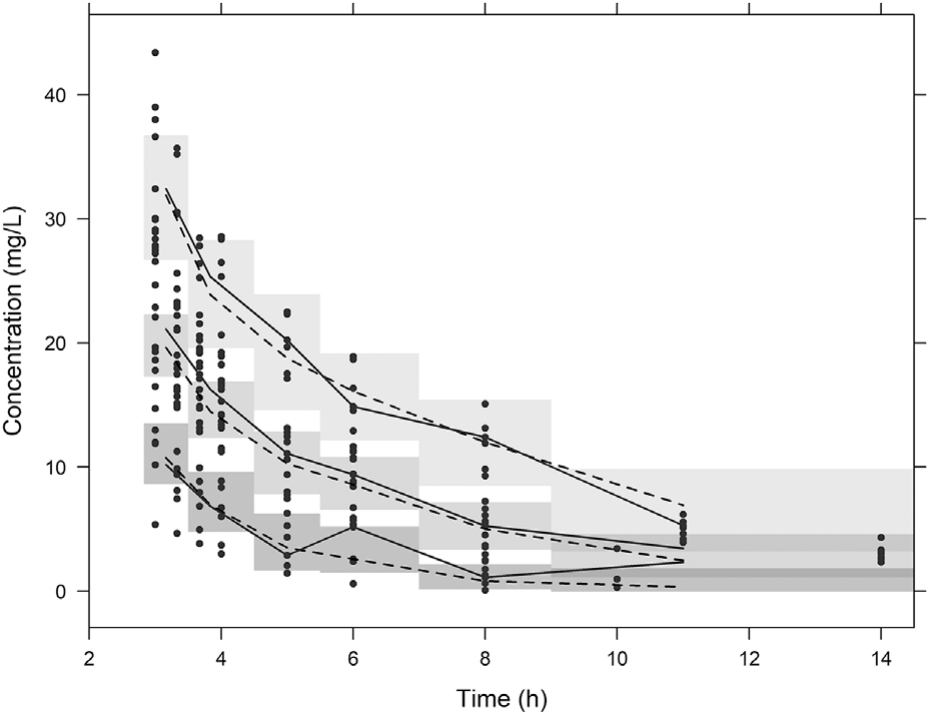
Visual predictive check plots. Plots from simulated concentrations of 1,000 virtual datasets: closed circles = observed concentrations; solid lines = 10th, 50th, and 90th percentiles of observations; dashed lines = 10th, 50th^,^ and 90th percentiles of simulated concentrations; shaded areas = 95% confidence intervals for the 10th, 50th, and 90th percentiles of simulated concentrations.

Diagnostic plots for the final PK model are presented in Figure 1. The conditional weighted residual values (CWRES) were evenly distributed around zero (Figure 1A,1B), indicating no major bias in the structural model. The observed concentrations were evenly distributed around the line of identity, indicating that there was no bias in the population parameters (Figure 1C) and the structural model was appropriate for most individuals (Figure 1D). A prediction- and variability-corrected visual predictive check is presented in Figure 2. This plot shows that 176 of the 210 observed concentrations (83.8%) fell within the 80% prediction intervals, and the observed 10th, 50th, and 90th percentiles fell within the 95% confidence intervals (CIs) of the simulated 10th, 50th, and 90th percentiles. These results suggest that the final PK model appropriately describes the observed data and has acceptable predictive performance.

### PD Target Attainment

**FIGURE 3:**
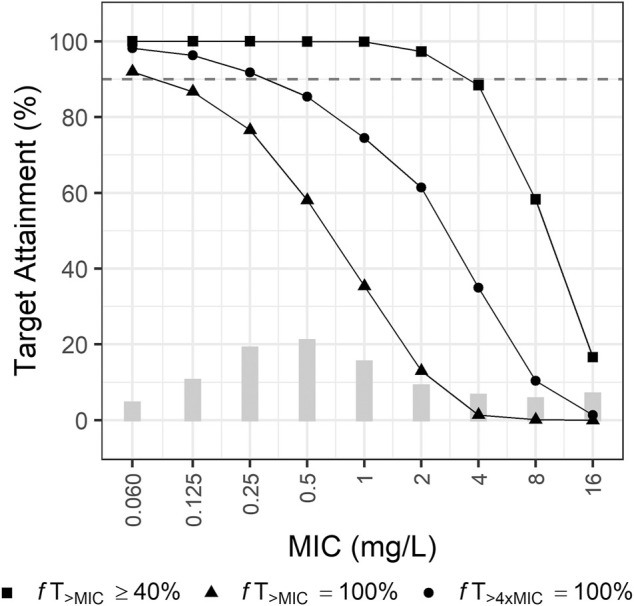
Probabilities of target attainment of empirical therapy by recommended dosing regimen for patients with creatinine clearance of 0–130 ml/min. Bars indicate the MIC distribution for *P. aeruginosa*.

The currently recommended dosing regimen for patients with intra-abdominal infection by *P. aeruginosa* was optimal when a target was 40% *f*T_>MIC_ and a MIC was equal to or less than 2 mg/L (Figure 3). When a MIC was 4 mg/L, the PTA was close to 90%, but not reached. For the target 100% *f*T_>MIC_, the recommended regimen was optimal when a MIC was equal to or less than 0.25 mg/L. For the target 100% *f*T_>4xMIC_, this regimen did not attain 90% when a MIC was greater than 0.125 mg/L.

**FIGURE 4:**
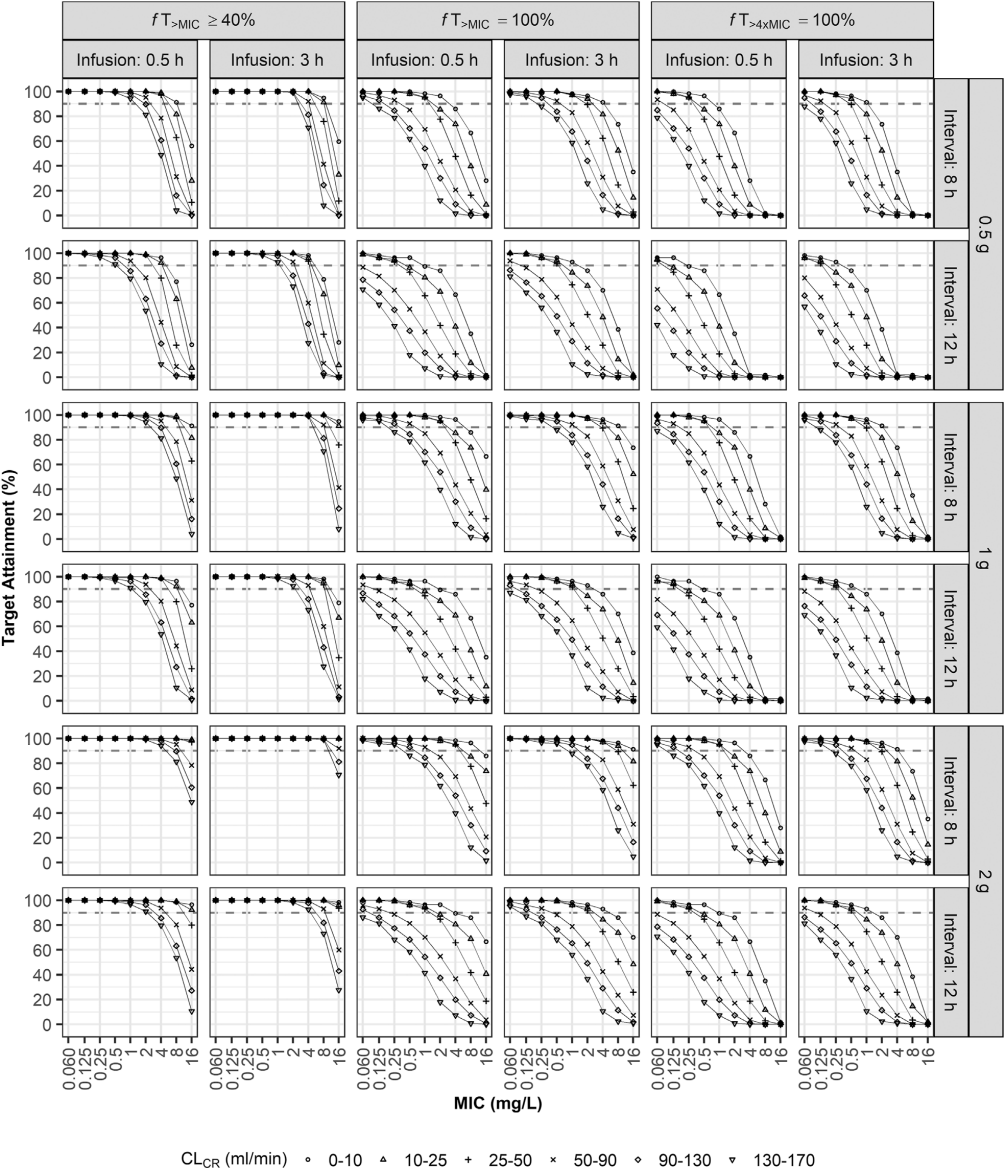
Probabilities of target attainment (40% *f*T_>_MIC, 100% *f*T_>_MIC, and 100% *f*T_>4xMIC_). Monte Carlo simulation results for virtual ECMO patients when using combinations of three doses (0.5, 1, or 2 g), two infusion times (0.5 or 3 h), two dosing intervals (8 or 12 h), and various MICs and degrees of renal impairment as model inputs.

The optimal dosage regimen for the three treatment targets was investigated intensively under various conditions (Figure 4; Supplementary Tables S1A–S1C). For patients with normal renal function (CL_CR_ of 90–130 ml/min), a dosing regimen of 1 g q8h by i. v. infusion over 0.5 h was optimal when the target was 40% *f*T_>MIC_ (Figure 4; Supplementary Table S1A) and the MIC was equal to 4 mg/L; 2 g q8h over 3 h was optimal when the target was 100% *f*T_>MIC_ (Figure 4; Supplementary Table S1B) and the MIC was equal to 1 mg/L; and 2 g q8h over 3 h was optimal when the target was 100% *f*T_>4xMIC_ (Figure 4; Supplementary Table S1C) and the MIC was less than 0.5 mg/L. For patients with CL_CR_ of 10–25 ml/min, a dosing regimen of 2 g q8h by i. v. infusion over 3 h was optimal when the target was 100% *f*T_>4xMIC_ and the MIC was equal to 2 mg/L. For patients with CL_CR_ of 25–50 ml/min, a dosing regimen of 0.5 g q8h by i. v. infusion over 0.5 h was optimal (PTA ≥90%) when the target was 40% *f*T_>MIC_ and the MIC was equal to 4 mg/L. For patients with CL_CR_ of 50–90 ml/min, a dosing regimen of 1 g q8h by i. v. infusion over 3 h was optimal when the target was 100% *f*T_>MIC_ and the MIC was equal to 1 mg/L.

The ECMO flow rate, the only significant covariate for V_C_, had a slight effect on the PTA (Supplementary Figure S2). For patients with CL_CR_ of 50–90 ml/min, a dosing regimen of 1 g q12h by i. v. infusion over 0.5 h was optimal for the MIC of 4 mg/L when the flow rate was 4 L/min or 6 L/min, while that was not optimal when the flow rate was 2 L/min.

## Discussion

In the present study, we used a dense sampling scheme to analyze the concentration-time profiles for IV meropenem infusion and better understand the population PK properties of meropenem in adult ECMO patients. To the best of our knowledge, this study was the largest prospective study to date: we investigated the PK/PD index (*f*T_>MIC_) for meropenem in 30 adult patients on ECMO. We could find only two population PK models of meropenem, one of which included 11 and the other 10 adult patients on ECMO (Shekar et al., 2014; Hanberg et al., 2018). As in the two previous studies of ECMO patients administered meropenem, the PK profile of meropenem in our study was described best by a two-compartment model. Typical model-predicted CL and steady-state volume of distribution (V_SS_ = V_C_ + V_P_) values for meropenem in the present study were 7.35 L/h and 30.1 L (V_C_ = 17.3 L and V_P_ = 12.8 L), respectively (Table 2). Individual model-predicted CL and V_SS_ are shown in Supplementary Table S2.

The findings of our study demonstrated a similar CL and a slightly increased V_SS_ in patients on ECMO compared to the values reported in a review article summarizing previous studies, in which the ranges of CL and V_SS_ for healthy volunteers were 11.2–19.8 L/h and 11.7–26.1 L, those for patients with mild to severe renal impairment were 2.0–7.7 L/h and 14.2–26.7 L, and those for patients with serious infection were 11.4–18.9 L/h and 20.7–26.7 L, respectively (Hurst and Lamb, 2000). Many factors can alter the volume of distribution in patients receiving ECMO therapy, including drug sequestration, hemodilution, hemodynamic physiologic changes, and systemic inflammation response syndrome (Sherwin et al., 2016; Cheng et al., 2018). Sequestration refers to binding of a drug to the ECMO circuit, which is more common for lipophilic or highly protein-bound drugs (Mousavi et al., 2011). However, since meropenem is a hydrophilic drug and the protein binding of meropenem is only about 2%, the V_SS_ does not appear to increase significantly when a patient is on EMCO therapy. Although a significant amount of meropenem was sequestered in the ECMO circuit after 4–6 h of treatment in an *ex vivo* experiment (Shekar et al., 2012), the actual effect is likely to be small *in vivo*, since meropenem was predicted to have a short half-life of 2.84 h in this study, and large amounts might be eliminated before sequestration.

In our study, CRRT patients demonstrated a reduced meropenem CL (6.03 L/h vs. 8.57 L/h) and an increased V_SS_ (35.4 vs. 25.0 L) when compared with non-CRRP patients (Table 3), although these trends were not statistically significant, as shown in previous population PK studies. In a matched cohort study of 11 ECMO patients and 10 non-ECMO patients, CL for patients with and without renal replacement therapy were 5.1 L/h and 9.6 L/h, respectively, and the V_SS_ was 32.9 L (V_C_ = 18.7 L and V_P_ = 13.2 L)(Shekar et al., 2014). In a more recent study that included 10 ECMO patients, 9 of which received renal replacement therapy, typical values of CL and V_SS_ were 2.79 L/h and 15.3 L (V_C_ = 8.31 L and V_P_ = 6.99 L), respectively (Hanberg et al., 2018). These two studies found that ECMO use did not have a statistically significant effect on meropenem PK. In our study, the ECMO flow rate was a significant covariate for V_C_. When the ECMO flow rate changed by 1 L, the V_C_ changed by 33.7%. The ECMO flow rate was expected to influence the CL or Q but was instead found to alter the V_C_. Hemodilution by administration of crystalloid fluids to maintain ECMO circuit flow might increase the V_C_ (Sherwin et al., 2016).

We implemented Monte Carlo simulations to evaluate the PTA when the treatment targets were, 100% *f*T_>MIC_ and 100% *f*T_>4xMIC_ as well as 40% *f*T_>MIC_ for meropenem, because the more aggressive targets of 100% *f*T_>MIC_ or 100% *f*T_>4xMIC_ has been suggested to improve clinical outcome for critically ill patients in intensive care unit (Roberts et al., 2014; Kothekar et al., 2020; Scharf et al., 2020). EUCAST epidemiological cut-off values (ECOFF) for gram-negative pathogens are 0.06 mg/L for Escherichia coli, 0.125 mg/L for Klebsiella pneumoniae. 2 mg/L for *P. aeruginosa*, and 4 mg/L for Acinetobacter baumannii (https://www.eucast.org/). The breakpoints are based on the standard dosage of meropenem (1 g q8h i. v. infusion over 30 min) and the high dosage (2 g q8h i. v. infusion over 3 h). For pathogens with reduced susceptibility exceeding a MIC of 2 mg/L, a dosage regimen different from that for wild-type pathogens may be required. Our simulation results show that the recommended dosage regimen provides sufficient meropenem concentration to empirically treat intra-abdominal infection by *P. aeruginosa* when the treatment target is 40% *f*T_>MIC_ and the MIC is equal to or less than 2 mg/L. However, if targets of 100% *f*T_>MIC_ and 100% *f*T_>4xMIC_ are adopted, dose increase may be required, though prolonged infusion times of 3 h were advantageous in achieving higher PTA (Figure 4; Supplementary Tables S1A–S1C). When the ECMO flow rate increased, the PTA tended to increase (Figure 4). When the volume of distribution was large, the plasma meropenem concentration was low, but the half-life became longer; these collectively increased the length of time the meropenem concentration remained above the MIC. Considering the complexity of conditions affecting the PK/PD index, therapeutic drug monitoring is recommended for patients undergoing ECMO in real-world clinical practice.

The present study had some limitations. First, our study did not include a control group of non-ECMO patients receiving meropenem. The results of this study had to be compared indirectly with the literature results, because it was not ethical to place patients who required ECMO support into a control group that did not use ECMO. Second, since a linear equation was used to address the covariate effect of ECMO flow rate on V_C_, V_C_ had a negative (non-physiological) value when the ECMO flow rate was less than 0.733 L/min. Therefore, care should be taken not to extrapolate this finding beyond the range of ECMO flow rates used in this analysis. Third, our simulations shows that an overdose or underdose is necessary depending on the situation. However, these results cannot be directly applied to clinical practice and more research is needed. Fourth, only the lower bound of the treatment target but not the toxicity level was considered when determining the PTA. When administering meropenem, it is desirable to choose the lowest total dose that attains 90% PTA. Fifth, we used doses and infusion times in the simulation, which were not actually included in our clinical study. Although extrapolation is often performed assuming PK linearity in many studies, it is necessary to pay attention to interpretation of the part outside the scope of the study.

## Conclusion

This study describes the meropenem PK profiles in adult patients on ECMO with a two-compartment model, in which CL_CR_ and the ECMO flow rate were significant covariates of CL and V_C_, respectively. Our simulation results provided an appropriate meropenem dosage regimen for patients on ECMO. The simulation predicted that if patients on ECMO are administered 1 g of meropenem q8h by i. v. infusion over 3 h, most of them can achieve the PK/PD target of 40% *f*T_>MIC._ when a MIC is equal to or less than 4 mg/L. However, dose increment and prolonged infusion may be necessary, considering the PK/PD target of 100% *f*T_>MIC_ or 100% *f*T_>4xMIC_ Since there are patients who do not reach the PK/PD target when applying the simulation results that addressed renal function, dosage regimens, and ECMO flow rate, we advocate therapeutic drug monitoring using a robust PK model to achieve precision dosing of meropenem.

## Data Availability

The raw data supporting the conclusions of this article will be made available by the authors, without undue reservation.
