# Supplementary figures and images for: Pharmacokinetics and Monte Carlo Simulation of Meropenem in Critically Ill Adult Patients Receiving Extracorporeal Membrane Oxygenation
Source: Front Pharmacol. 2021 Nov 1;12:768912. doi: 10.3389/fphar.2021.768912 (PMC8591204; doi:10.3389/fphar.2021.768912)

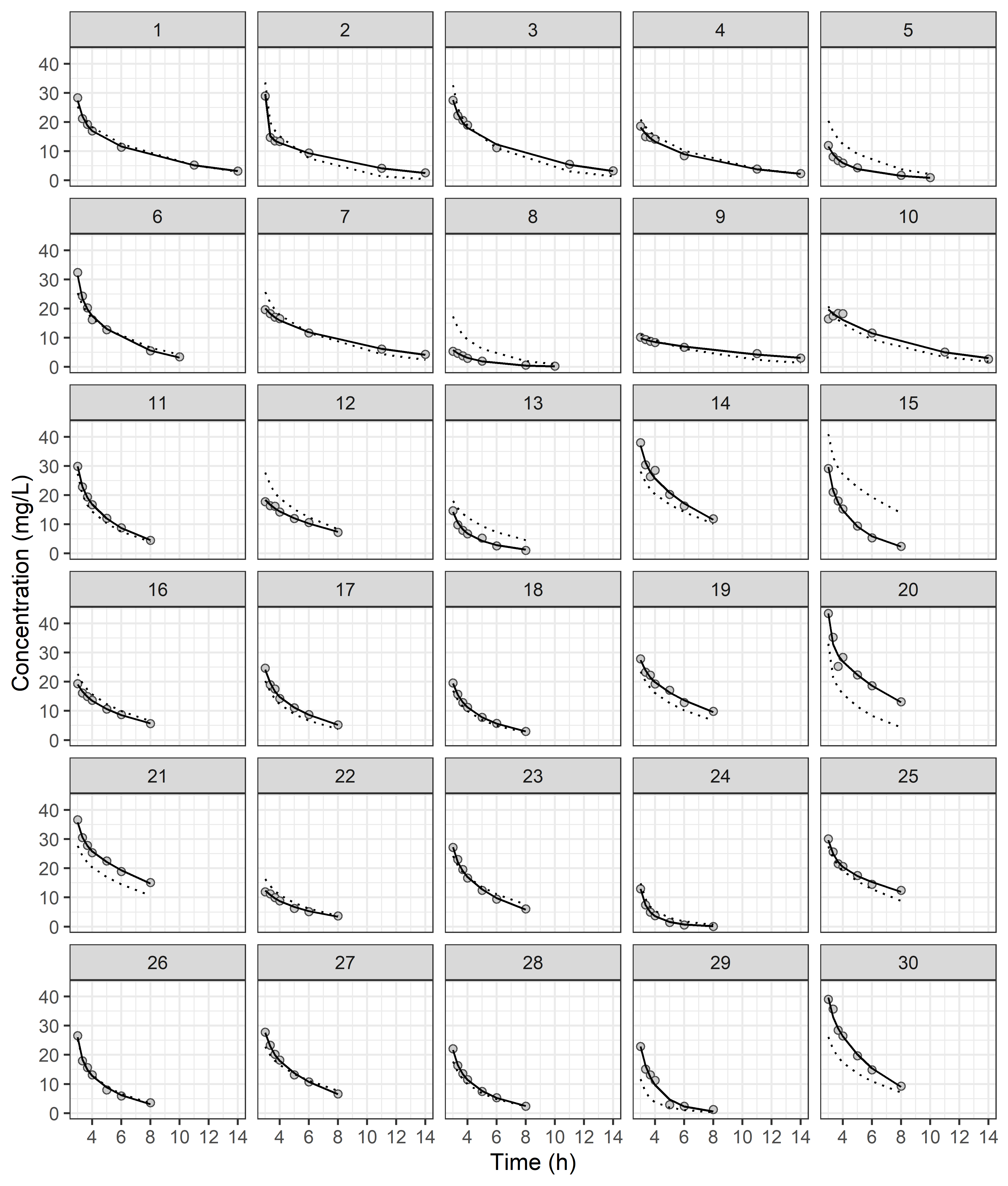

Supplement: Supplementary file 1 [file Image1.TIFF]

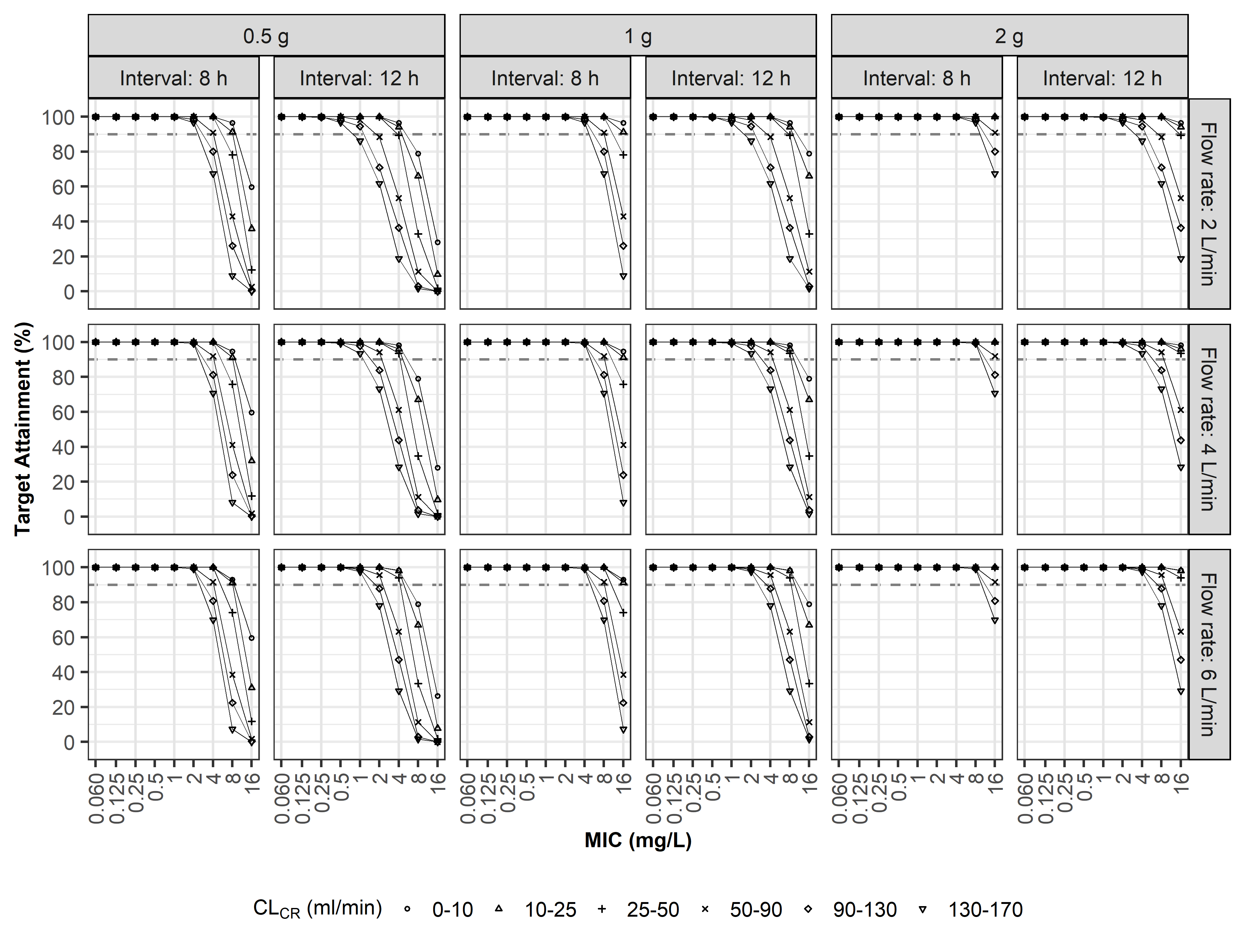

Supplement: Supplementary file 2 [file Image2.TIFF]
