# Supplementary material for: Pharmacokinetics and Monte Carlo Simulation of Meropenem in Critically Ill Adult Patients Receiving Extracorporeal Membrane Oxygenation
Source: Front Pharmacol. 2021 Nov 1;12:768912. doi: 10.3389/fphar.2021.768912 (PMC8591204; doi:10.3389/fphar.2021.768912)
Supplement: Supplementary file 3 [file DataSheet1.docx]

Supplementary Material

# Supplementary Tables

**Supplementary Table 1A. Probability of target attainment of 40% *f*T_>MIC_ with degrees of renal function, various regimen, and MIC**

| **MIC (mg/L)** | **0.06** | **0.13** | **0.25** | **0.5** | **1** | **2** | **4** | **8** | **16** |  | **0.06** | **0.13** | **0.25** | **0.5** | **1** | **2** | **4** | **8** | **16** |
| --- | --- | --- | --- | --- | --- | --- | --- | --- | --- | --- | --- | --- | --- | --- | --- | --- | --- | --- | --- |
| **CL_CR_ (ml/min)** | **500 mg q8h & 0.5 h infusion** | | | | | | | | |  | **500 mg q12h & 0.5 h infusion** | | | | | | | | |
| 0-10 | 100 | 100 | 100 | 100 | 100 | 100 | 98 | 91 | 56 |  | 100 | 100 | 100 | 100 | 100 | 98 | 96 | 77 | 26 |
| 10-25 | 100 | 100 | 100 | 100 | 100 | 100 | 99 | 82 | 28 |  | 100 | 100 | 100 | 100 | 100 | 99 | 92 | 63 | 8 |
| 25-50 | 100 | 100 | 100 | 100 | 100 | 100 | 97 | 63 | 11 |  | 100 | 100 | 100 | 100 | 100 | 99 | 80 | 26 | 2 |
| 50-90 | 100 | 100 | 100 | 100 | 100 | 95 | 79 | 31 | 2 |  | 100 | 100 | 100 | 99 | 94 | 80 | 44 | 9 | 0 |
| 90-130 | 100 | 100 | 100 | 100 | 98 | 90 | 61 | 16 | 0 |  | 100 | 100 | 99 | 96 | 86 | 63 | 27 | 1 | 0 |
| 130-170 | 100 | 100 | 100 | 99 | 94 | 81 | 49 | 4 | 0 |  | 100 | 99 | 97 | 91 | 80 | 54 | 11 | 1 | 0 |
| **CL_CR_ (ml/min)** | **500 mg q8h & 3 h infusion** | | | | | | | | |  | **500 mg q12h & 3 h infusion** | | | | | | | | |
| 0-10 | 100 | 100 | 100 | 100 | 100 | 100 | 100 | 95 | 60 |  | 100 | 100 | 100 | 100 | 100 | 100 | 98 | 79 | 28 |
| 10-25 | 100 | 100 | 100 | 100 | 100 | 100 | 100 | 91 | 33 |  | 100 | 100 | 100 | 100 | 100 | 100 | 96 | 67 | 10 |
| 25-50 | 100 | 100 | 100 | 100 | 100 | 100 | 100 | 76 | 12 |  | 100 | 100 | 100 | 100 | 100 | 100 | 94 | 35 | 2 |
| 50-90 | 100 | 100 | 100 | 100 | 100 | 100 | 92 | 41 | 2 |  | 100 | 100 | 100 | 100 | 100 | 93 | 60 | 11 | 0 |
| 90-130 | 100 | 100 | 100 | 100 | 100 | 99 | 81 | 25 | 0 |  | 100 | 100 | 100 | 99 | 98 | 82 | 43 | 3 | 0 |
| 130-170 | 100 | 100 | 100 | 100 | 100 | 99 | 71 | 8 | 0 |  | 100 | 100 | 100 | 98 | 93 | 73 | 28 | 2 | 0 |
| **CL_CR_ (ml/min)** | **1000 mg q8h & 0.5 h infusion** | | | | | | | | |  | **1000 mg q12h & 0.5 h infusion** | | | | | | | | |
| 0-10 | 100 | 100 | 100 | 100 | 100 | 100 | 100 | 98 | 91 |  | 100 | 100 | 100 | 100 | 100 | 100 | 98 | 96 | 77 |
| 10-25 | 100 | 100 | 100 | 100 | 100 | 100 | 100 | 99 | 82 |  | 100 | 100 | 100 | 100 | 100 | 100 | 99 | 92 | 63 |
| 25-50 | 100 | 100 | 100 | 100 | 100 | 100 | 100 | 97 | 63 |  | 100 | 100 | 100 | 100 | 100 | 100 | 99 | 80 | 26 |
| 50-90 | 100 | 100 | 100 | 100 | 100 | 100 | 95 | 79 | 31 |  | 100 | 100 | 100 | 100 | 99 | 94 | 80 | 44 | 9 |
| 90-130 | 100 | 100 | 100 | 100 | 100 | 98 | 90 | 61 | 16 |  | 100 | 100 | 100 | 99 | 96 | 86 | 63 | 27 | 1 |
| 130-170 | 100 | 100 | 100 | 100 | 99 | 94 | 81 | 49 | 4 |  | 100 | 100 | 99 | 97 | 91 | 80 | 54 | 11 | 1 |
| **CL_CR_ (ml/min)** | **1000 mg q8h & 3 h infusion** | | | | | | | | |  | **1000 mg q12h & 3 h infusion** | | | | | | | | |
| 0-10 | 100 | 100 | 100 | 100 | 100 | 100 | 100 | 100 | 95 |  | 100 | 100 | 100 | 100 | 100 | 100 | 100 | 98 | 79 |
| 10-25 | 100 | 100 | 100 | 100 | 100 | 100 | 100 | 100 | 91 |  | 100 | 100 | 100 | 100 | 100 | 100 | 100 | 96 | 67 |
| 25-50 | 100 | 100 | 100 | 100 | 100 | 100 | 100 | 100 | 76 |  | 100 | 100 | 100 | 100 | 100 | 100 | 100 | 94 | 35 |
| 50-90 | 100 | 100 | 100 | 100 | 100 | 100 | 100 | 92 | 41 |  | 100 | 100 | 100 | 100 | 100 | 100 | 93 | 60 | 11 |
| 90-130 | 100 | 100 | 100 | 100 | 100 | 100 | 99 | 81 | 25 |  | 100 | 100 | 100 | 100 | 99 | 98 | 82 | 43 | 3 |
| 130-170 | 100 | 100 | 100 | 100 | 100 | 100 | 99 | 71 | 8 |  | 100 | 100 | 100 | 100 | 98 | 93 | 73 | 28 | 2 |
| **CL_CR_ (ml/min)** | **2000 mg q8h & 0.5 h infusion** | | | | | | | | |  | **2000 mg q12h & 0.5 h infusion** | | | | | | | | |
| 0-10 | 100 | 100 | 100 | 100 | 100 | 100 | 100 | 100 | 98 |  | 100 | 100 | 100 | 100 | 100 | 100 | 100 | 98 | 96 |
| 10-25 | 100 | 100 | 100 | 100 | 100 | 100 | 100 | 100 | 99 |  | 100 | 100 | 100 | 100 | 100 | 100 | 100 | 99 | 92 |
| 25-50 | 100 | 100 | 100 | 100 | 100 | 100 | 100 | 100 | 97 |  | 100 | 100 | 100 | 100 | 100 | 100 | 100 | 99 | 80 |
| 50-90 | 100 | 100 | 100 | 100 | 100 | 100 | 100 | 95 | 79 |  | 100 | 100 | 100 | 100 | 100 | 99 | 94 | 80 | 44 |
| 90-130 | 100 | 100 | 100 | 100 | 100 | 100 | 98 | 90 | 61 |  | 100 | 100 | 100 | 100 | 99 | 96 | 86 | 63 | 27 |
| 130-170 | 100 | 100 | 100 | 100 | 100 | 99 | 94 | 81 | 49 |  | 100 | 100 | 100 | 99 | 97 | 91 | 80 | 54 | 11 |
| **CL_CR_ (ml/min)** | **2000 mg q8h & 3 h infusion** | | | | | | | | |  | **2000 mg q12h & 3 h infusion** | | | | | | | | |
| 0-10 | 100 | 100 | 100 | 100 | 100 | 100 | 100 | 100 | 100 |  | 100 | 100 | 100 | 100 | 100 | 100 | 100 | 100 | 98 |
| 10-25 | 100 | 100 | 100 | 100 | 100 | 100 | 100 | 100 | 100 |  | 100 | 100 | 100 | 100 | 100 | 100 | 100 | 100 | 96 |
| 25-50 | 100 | 100 | 100 | 100 | 100 | 100 | 100 | 100 | 100 |  | 100 | 100 | 100 | 100 | 100 | 100 | 100 | 100 | 94 |
| 50-90 | 100 | 100 | 100 | 100 | 100 | 100 | 100 | 100 | 92 |  | 100 | 100 | 100 | 100 | 100 | 100 | 100 | 93 | 60 |
| 90-130 | 100 | 100 | 100 | 100 | 100 | 100 | 100 | 99 | 81 |  | 100 | 100 | 100 | 100 | 100 | 99 | 98 | 82 | 43 |
| 130-170 | 100 | 100 | 100 | 100 | 100 | 100 | 100 | 99 | 71 |  | 100 | 100 | 100 | 100 | 100 | 98 | 93 | 73 | 28 |

**Supplementary Table 1B. Probability of target attainment of 100% *f*T_>MIC_ with degrees of renal function, various regimen, and MIC**

| **MIC (mg/L)** | **0.06** | **0.13** | **0.25** | **0.5** | **1** | **2** | **4** | **8** | **16** |  | **0.06** | **0.13** | **0.25** | **0.5** | **1** | **2** | **4** | **8** | **16** |
| --- | --- | --- | --- | --- | --- | --- | --- | --- | --- | --- | --- | --- | --- | --- | --- | --- | --- | --- | --- |
| **CL_CR_ (ml/min)** | **500 mg q8h & 0.5 h infusion** | | | | | | | | |  | **500 mg q12h & 0.5 h infusion** | | | | | | | | |
| 0-10 | 100 | 100 | 100 | 100 | 98 | 96 | 86 | 67 | 28 |  | 100 | 98 | 96 | 96 | 89 | 86 | 67 | 35 | 2 |
| 10-25 | 100 | 100 | 100 | 98 | 95 | 85 | 74 | 40 | 9 |  | 99 | 96 | 95 | 88 | 81 | 69 | 41 | 12 | 1 |
| 25-50 | 100 | 100 | 100 | 99 | 95 | 78 | 48 | 16 | 1 |  | 100 | 96 | 93 | 85 | 66 | 42 | 19 | 3 | 0 |
| 50-90 | 99 | 97 | 93 | 85 | 69 | 44 | 21 | 4 | 0 |  | 89 | 81 | 70 | 55 | 37 | 17 | 4 | 0 | 0 |
| 90-130 | 97 | 93 | 85 | 70 | 54 | 30 | 9 | 0 | 0 |  | 79 | 68 | 54 | 38 | 20 | 7 | 1 | 0 | 0 |
| 130-170 | 95 | 86 | 79 | 62 | 40 | 12 | 2 | 0 | 0 |  | 71 | 59 | 41 | 18 | 7 | 1 | 0 | 0 | 0 |
| **CL_CR_ (ml/min)** | **500 mg q8h & 3 h infusion** | | | | | | | | |  | **500 mg q12h & 3 h infusion** | | | | | | | | |
| 0-10 | 100 | 100 | 100 | 100 | 98 | 96 | 91 | 74 | 35 |  | 100 | 100 | 98 | 96 | 93 | 86 | 70 | 39 | 2 |
| 10-25 | 100 | 100 | 100 | 100 | 97 | 94 | 82 | 52 | 15 |  | 100 | 99 | 96 | 94 | 84 | 74 | 49 | 15 | 2 |
| 25-50 | 100 | 100 | 100 | 100 | 98 | 89 | 62 | 25 | 3 |  | 100 | 100 | 96 | 92 | 75 | 51 | 26 | 4 | 0 |
| 50-90 | 100 | 99 | 97 | 92 | 83 | 59 | 31 | 8 | 0 |  | 94 | 88 | 79 | 65 | 43 | 24 | 7 | 0 | 0 |
| 90-130 | 99 | 97 | 95 | 84 | 68 | 44 | 17 | 1 | 0 |  | 86 | 76 | 65 | 49 | 29 | 11 | 2 | 0 | 0 |
| 130-170 | 98 | 96 | 88 | 78 | 59 | 26 | 5 | 1 | 0 |  | 81 | 68 | 56 | 37 | 11 | 2 | 1 | 0 | 0 |
| **CL_CR_ (ml/min)** | **1000 mg q8h & 0.5 h infusion** | | | | | | | | |  | **1000 mg q12h & 0.5 h infusion** | | | | | | | | |
| 0-10 | 100 | 100 | 100 | 100 | 100 | 98 | 96 | 86 | 67 |  | 100 | 100 | 98 | 96 | 96 | 89 | 86 | 67 | 35 |
| 10-25 | 100 | 100 | 100 | 100 | 98 | 95 | 85 | 74 | 40 |  | 100 | 99 | 96 | 95 | 88 | 81 | 69 | 41 | 12 |
| 25-50 | 100 | 100 | 100 | 100 | 99 | 95 | 78 | 48 | 16 |  | 100 | 100 | 96 | 93 | 85 | 66 | 42 | 19 | 3 |
| 50-90 | 99 | 99 | 97 | 93 | 85 | 69 | 44 | 21 | 4 |  | 93 | 89 | 81 | 70 | 55 | 37 | 17 | 4 | 0 |
| 90-130 | 98 | 96 | 93 | 85 | 70 | 54 | 30 | 9 | 0 |  | 87 | 78 | 68 | 54 | 38 | 20 | 7 | 1 | 0 |
| 130-170 | 96 | 95 | 86 | 79 | 62 | 40 | 12 | 2 | 0 |  | 82 | 68 | 59 | 41 | 18 | 7 | 1 | 0 | 0 |
| **CL_CR_ (ml/min)** | **1000 mg q8h & 3 h infusion** | | | | | | | | |  | **1000 mg q12h & 3 h infusion** | | | | | | | | |
| 0-10 | 100 | 100 | 100 | 100 | 100 | 98 | 96 | 91 | 74 |  | 100 | 100 | 100 | 98 | 96 | 93 | 86 | 70 | 39 |
| 10-25 | 100 | 100 | 100 | 100 | 100 | 97 | 94 | 82 | 52 |  | 100 | 100 | 99 | 96 | 94 | 84 | 74 | 49 | 15 |
| 25-50 | 100 | 100 | 100 | 100 | 100 | 98 | 89 | 62 | 25 |  | 100 | 100 | 100 | 96 | 92 | 75 | 51 | 26 | 4 |
| 50-90 | 100 | 100 | 99 | 97 | 92 | 83 | 59 | 31 | 8 |  | 96 | 93 | 88 | 79 | 65 | 43 | 24 | 7 | 0 |
| 90-130 | 99 | 99 | 97 | 95 | 84 | 68 | 44 | 17 | 1 |  | 93 | 86 | 76 | 65 | 49 | 29 | 11 | 2 | 0 |
| 130-170 | 99 | 97 | 96 | 88 | 78 | 59 | 26 | 5 | 1 |  | 87 | 81 | 68 | 56 | 37 | 11 | 2 | 1 | 0 |
| **CL_CR_ (ml/min)** | **2000 mg q8h & 0.5 h infusion** | | | | | | | | |  | **2000 mg q12h & 0.5 h infusion** | | | | | | | | |
| 0-10 | 100 | 100 | 100 | 100 | 100 | 100 | 98 | 96 | 86 |  | 100 | 100 | 100 | 98 | 96 | 96 | 89 | 86 | 67 |
| 10-25 | 100 | 100 | 100 | 100 | 100 | 98 | 95 | 85 | 74 |  | 100 | 100 | 99 | 96 | 95 | 88 | 81 | 69 | 41 |
| 25-50 | 100 | 100 | 100 | 100 | 100 | 99 | 95 | 78 | 48 |  | 100 | 100 | 100 | 96 | 93 | 85 | 66 | 42 | 19 |
| 50-90 | 100 | 99 | 99 | 97 | 93 | 85 | 69 | 44 | 21 |  | 96 | 93 | 89 | 81 | 70 | 55 | 37 | 17 | 4 |
| 90-130 | 99 | 98 | 96 | 93 | 85 | 70 | 54 | 30 | 9 |  | 93 | 86 | 78 | 68 | 54 | 38 | 20 | 7 | 1 |
| 130-170 | 98 | 96 | 95 | 86 | 79 | 62 | 40 | 12 | 2 |  | 86 | 81 | 68 | 59 | 41 | 18 | 7 | 1 | 0 |
| **CL_CR_ (ml/min)** | **2000 mg q8h & 3 h infusion** | | | | | | | | |  | **2000 mg q12h & 3 h infusion** | | | | | | | | |
| 0-10 | 100 | 100 | 100 | 100 | 100 | 100 | 98 | 96 | 91 |  | 100 | 100 | 100 | 100 | 98 | 96 | 93 | 86 | 70 |
| 10-25 | 100 | 100 | 100 | 100 | 100 | 100 | 97 | 94 | 82 |  | 100 | 100 | 100 | 99 | 96 | 94 | 84 | 74 | 49 |
| 25-50 | 100 | 100 | 100 | 100 | 100 | 100 | 98 | 89 | 62 |  | 100 | 100 | 100 | 100 | 96 | 92 | 75 | 51 | 26 |
| 50-90 | 100 | 100 | 100 | 99 | 97 | 92 | 83 | 59 | 31 |  | 99 | 96 | 93 | 88 | 79 | 65 | 43 | 24 | 7 |
| 90-130 | 100 | 99 | 99 | 97 | 95 | 84 | 68 | 44 | 17 |  | 96 | 93 | 86 | 76 | 65 | 49 | 29 | 11 | 2 |
| 130-170 | 100 | 99 | 97 | 96 | 88 | 78 | 59 | 26 | 5 |  | 95 | 87 | 81 | 68 | 56 | 37 | 11 | 2 | 1 |

**Supplementary Table 1C. Probability of target attainment of 100% *f*T_>4xMIC_ with degrees of renal function, various regimen, and MIC**

| **MIC (mg/L)** | **0.06** | **0.13** | **0.25** | **0.5** | **1** | **2** | **4** | **8** | **16** |  | **0.06** | **0.13** | **0.25** | **0.5** | **1** | **2** | **4** | **8** | **16** |
| --- | --- | --- | --- | --- | --- | --- | --- | --- | --- | --- | --- | --- | --- | --- | --- | --- | --- | --- | --- |
| **CL_CR_ (ml/min)** | **500 mg q8h & 0.5 h infusion** | | | | | | | | |  | **500 mg q12h & 0.5 h infusion** | | | | | | | | |
| 0-10 | 100 | 100 | 98 | 96 | 86 | 67 | 28 | 2 | 0 |  | 96 | 96 | 89 | 86 | 67 | 35 | 2 | 2 | 0 |
| 10-25 | 100 | 98 | 95 | 85 | 74 | 40 | 9 | 1 | 0 |  | 95 | 88 | 81 | 69 | 41 | 12 | 1 | 0 | 0 |
| 25-50 | 100 | 99 | 95 | 78 | 48 | 16 | 1 | 0 | 0 |  | 94 | 85 | 66 | 42 | 19 | 3 | 0 | 0 | 0 |
| 50-90 | 93 | 85 | 69 | 44 | 21 | 4 | 0 | 0 | 0 |  | 71 | 55 | 37 | 17 | 4 | 0 | 0 | 0 | 0 |
| 90-130 | 85 | 70 | 54 | 30 | 9 | 0 | 0 | 0 | 0 |  | 56 | 38 | 20 | 7 | 1 | 0 | 0 | 0 | 0 |
| 130-170 | 79 | 62 | 40 | 12 | 2 | 0 | 0 | 0 | 0 |  | 42 | 18 | 7 | 1 | 0 | 0 | 0 | 0 | 0 |
| **CL_CR_ (ml/min)** | **500 mg q8h & 3 h infusion** | | | | | | | | |  | **500 mg q12h & 3 h infusion** | | | | | | | | |
| 0-10 | 100 | 100 | 98 | 96 | 91 | 74 | 35 | 2 | 0 |  | 98 | 96 | 93 | 86 | 70 | 39 | 2 | 2 | 0 |
| 10-25 | 100 | 100 | 97 | 94 | 82 | 52 | 15 | 1 | 0 |  | 96 | 94 | 84 | 74 | 49 | 15 | 2 | 0 | 0 |
| 25-50 | 100 | 100 | 98 | 89 | 62 | 25 | 3 | 0 | 0 |  | 96 | 92 | 75 | 51 | 26 | 4 | 0 | 0 | 0 |
| 50-90 | 97 | 92 | 83 | 59 | 31 | 8 | 0 | 0 | 0 |  | 80 | 65 | 43 | 24 | 7 | 0 | 0 | 0 | 0 |
| 90-130 | 95 | 84 | 68 | 44 | 17 | 1 | 0 | 0 | 0 |  | 66 | 49 | 29 | 11 | 2 | 0 | 0 | 0 | 0 |
| 130-170 | 88 | 78 | 59 | 26 | 5 | 1 | 0 | 0 | 0 |  | 57 | 37 | 11 | 2 | 1 | 0 | 0 | 0 | 0 |
| **CL_CR_ (ml/min)** | **1000 mg q8h & 0.5 h infusion** | | | | | | | | |  | **1000 mg q12h & 0.5 h infusion** | | | | | | | | |
| 0-10 | 100 | 100 | 100 | 98 | 96 | 86 | 67 | 28 | 2 |  | 100 | 96 | 96 | 89 | 86 | 67 | 35 | 2 | 2 |
| 10-25 | 100 | 100 | 98 | 95 | 85 | 74 | 40 | 9 | 1 |  | 96 | 95 | 88 | 81 | 69 | 41 | 12 | 1 | 0 |
| 25-50 | 100 | 100 | 99 | 95 | 78 | 48 | 16 | 1 | 0 |  | 97 | 93 | 85 | 66 | 42 | 19 | 3 | 0 | 0 |
| 50-90 | 97 | 93 | 85 | 69 | 44 | 21 | 4 | 0 | 0 |  | 82 | 70 | 55 | 37 | 17 | 4 | 0 | 0 | 0 |
| 90-130 | 93 | 85 | 70 | 54 | 30 | 9 | 0 | 0 | 0 |  | 69 | 54 | 38 | 20 | 7 | 1 | 0 | 0 | 0 |
| 130-170 | 87 | 79 | 62 | 40 | 12 | 2 | 0 | 0 | 0 |  | 59 | 41 | 18 | 7 | 1 | 0 | 0 | 0 | 0 |
| **CL_CR_ (ml/min)** | **1000 mg q8h & 3 h infusion** | | | | | | | | |  | **1000 mg q12h & 3 h infusion** | | | | | | | | |
| 0-10 | 100 | 100 | 100 | 98 | 96 | 91 | 74 | 35 | 2 |  | 100 | 98 | 96 | 93 | 86 | 70 | 39 | 2 | 2 |
| 10-25 | 100 | 100 | 100 | 97 | 94 | 82 | 52 | 15 | 1 |  | 99 | 96 | 94 | 84 | 74 | 49 | 15 | 2 | 0 |
| 25-50 | 100 | 100 | 100 | 98 | 89 | 62 | 25 | 3 | 0 |  | 100 | 96 | 92 | 75 | 51 | 26 | 4 | 0 | 0 |
| 50-90 | 99 | 97 | 92 | 83 | 59 | 31 | 8 | 0 | 0 |  | 88 | 79 | 65 | 43 | 24 | 7 | 0 | 0 | 0 |
| 90-130 | 97 | 95 | 84 | 68 | 44 | 17 | 1 | 0 | 0 |  | 76 | 65 | 49 | 29 | 11 | 2 | 0 | 0 | 0 |
| 130-170 | 96 | 88 | 78 | 59 | 26 | 5 | 1 | 0 | 0 |  | 69 | 56 | 37 | 11 | 2 | 1 | 0 | 0 | 0 |
| **CL_CR_ (ml/min)** | **2000 mg q8h & 0.5 h infusion** | | | | | | | | |  | **2000 mg q12h & 0.5 h infusion** | | | | | | | | |
| 0-10 | 100 | 100 | 100 | 100 | 98 | 96 | 86 | 67 | 28 |  | 100 | 98 | 96 | 96 | 89 | 86 | 67 | 35 | 2 |
| 10-25 | 100 | 100 | 100 | 98 | 95 | 85 | 74 | 40 | 9 |  | 99 | 96 | 95 | 88 | 81 | 69 | 41 | 12 | 1 |
| 25-50 | 100 | 100 | 100 | 99 | 95 | 78 | 48 | 16 | 1 |  | 100 | 96 | 93 | 85 | 66 | 42 | 19 | 3 | 0 |
| 50-90 | 99 | 97 | 93 | 85 | 69 | 44 | 21 | 4 | 0 |  | 89 | 81 | 70 | 55 | 37 | 17 | 4 | 0 | 0 |
| 90-130 | 97 | 93 | 85 | 70 | 54 | 30 | 9 | 0 | 0 |  | 79 | 68 | 54 | 38 | 20 | 7 | 1 | 0 | 0 |
| 130-170 | 95 | 86 | 79 | 62 | 40 | 12 | 2 | 0 | 0 |  | 71 | 59 | 41 | 18 | 7 | 1 | 0 | 0 | 0 |
| **CL_CR_ (ml/min)** | **2000 mg q8h & 3 h infusion** | | | | | | | | |  | **2000 mg q12h & 3 h infusion** | | | | | | | | |
| 0-10 | 100 | 100 | 100 | 100 | 98 | 96 | 91 | 74 | 35 |  | 100 | 100 | 98 | 96 | 93 | 86 | 70 | 39 | 2 |
| 10-25 | 100 | 100 | 100 | 100 | 97 | 94 | 82 | 52 | 15 |  | 100 | 99 | 96 | 94 | 84 | 74 | 49 | 15 | 2 |
| 25-50 | 100 | 100 | 100 | 100 | 98 | 89 | 62 | 25 | 3 |  | 100 | 100 | 96 | 92 | 75 | 51 | 26 | 4 | 0 |
| 50-90 | 100 | 99 | 97 | 92 | 83 | 59 | 31 | 8 | 0 |  | 94 | 88 | 79 | 65 | 43 | 24 | 7 | 0 | 0 |
| 90-130 | 99 | 97 | 95 | 84 | 68 | 44 | 17 | 1 | 0 |  | 86 | 76 | 65 | 49 | 29 | 11 | 2 | 0 | 0 |
| 130-170 | 98 | 96 | 88 | 78 | 59 | 26 | 5 | 1 | 0 |  | 81 | 68 | 56 | 37 | 11 | 2 | 1 | 0 | 0 |

**Supplementary Table 2. Individual model-predicted total clearance and volume of distribution^a^**

| ID | CL  (L/h) | CL_CR_  (mg/dL) | CG  (nL/min) | mMDRD (mL/min) | mCE (mL/min) | V_C_  (L) | V_P_  (L) | V_SS_  (L) | ECMO flow rate  (L/min) | CRRT |
| --- | --- | --- | --- | --- | --- | --- | --- | --- | --- | --- |
| 1 | 5.79 | 1.81 | 31.8 | 27.3 | 28.0 | 12.2 | 18.6 | 30.7 | 3.96 | Yes |
| 2 | 6.20 | 2.54 | 49.5 | 33.0 | 35.4 | 2.57 | 25.5 | 28.0 | 1.27 | Yes |
| 3 | 5.66 | 2.01 | 35.2 | 35.2 | 38.6 | 13.5 | 16.5 | 30.0 | 2.09 | No |
| 4 | 8.24 | 1.85 | 51.4 | 44.1 | 44.8 | 29.4 | 15.1 | 44.6 | 5.00 | Yes |
| 5 | 20.3 | 0.76 | 89.2 | 103 | 98.0 | 30.1 | 22.7 | 52.9 | 4.00 | No |
| 6 | 6.85 | 1.06 | 47.2 | 58.9 | 58.8 | 8.04 | 12.4 | 20.5 | 3.50 | No |
| 7 | 5.86 | 1.64 | 37.3 | 29.5 | 31.1 | 29.8 | 13.3 | 43.1 | 3.70 | Yes |
| 8 | 45.1 | 0.53 | 137 | 155 | 118 | 72.3 | 15.5 | 87.8 | 4.04 | No |
| 9 | 3.89 | 3.52 | 19.6 | 19.2 | 17.8 | 15.6 | 19.9 | 35.5 | 2.66 | No |
| 10 | 6.76 | 2.30 | 59.1 | 39.1 | 40.7 | 33.2 | 7.08 | 40.2 | 4.78 | Yes |
| 11 | 7.57 | 0.99 | 63.3 | 75.2 | 78.6 | 9.30 | 10.9 | 20.1 | 2.30 | No |
| 12 | 7.33 | 2.32 | 31.8 | 29.8 | 31.4 | 32.5 | 12.0 | 44.5 | 3.30 | Yes |
| 13 | 18.1 | 0.72 | 88.2 | 80.7 | 88.9 | 21.0 | 15.2 | 36.2 | 5.34 | No |
| 14 | 4.42 | 3.11 | 21.1 | 15.2 | 15.1 | 10.4 | 10.3 | 20.7 | 3.54 | Yes |
| 15 | 8.89 | 21.7 | 1.91 | 1.86 | 1.52 | 8.13 | 8.25 | 16.4 | 2.00 | No |
| 16 | 8.85 | 0.91 | 52.9 | 57.4 | 57.5 | 26.3 | 13.4 | 39.7 | 4.21 | Yes |
| 17 | 8.24 | 0.73 | 90.8 | 111 | 94.5 | 14.8 | 13.7 | 28.6 | 4.00 | No |
| 18 | 11.7 | 0.56 | 130 | 116 | 107 | 18.5 | 12.7 | 31.3 | 4.70 | No |
| 19 | 5.48 | 1.05 | 50.0 | 49.7 | 53.3 | 17.1 | 12.2 | 29.2 | 4.03 | Yes |
| 20 | 3.79 | 1.72 | 47.3 | 35.8 | 39.7 | 5.20 | 13.2 | 18.3 | 1.50 | No |
| 21 | 3.54 | 1.65 | 19.2 | 23.8 | 22.7 | 11.4 | 13.2 | 24.6 | 3.70 | No |
| 22 | 14.3 | 1.73 | 111 | 70.5 | 76.9 | 44.1 | 15.6 | 59.7 | 5.75 | No |
| 23 | 7.36 | 1.35 | 42.3 | 51.4 | 49.8 | 13.0 | 11.8 | 24.9 | 4.00 | No |
| 24 | 24.2 | 0.63 | 208 | 170 | 150 | 12.1 | 9.21 | 21.4 | 2.99 | No |
| 25 | 4.23 | 1.37 | 29.5 | 32.0 | 31.8 | 13.5 | 17.6 | 31.0 | 3.44 | Yes |
| 26 | 9.39 | 0.69 | 75.8 | 84.0 | 84.0 | 8.5 | 12.5 | 21.0 | 2.48 | No |
| 27 | 6.94 | 1.37 | 42.2 | 39.2 | 38.8 | 14.1 | 11.2 | 25.3 | 4.50 | No |
| 28 | 11.3 | 0.72 | 127 | 129 | 117 | 13.8 | 11.5 | 25.2 | 4.20 | No |
| 29 | 13.1 | 0.24 | 290 | 304 | 140 | 11.1 | 5.2 | 16.3 | 3.54 | No |
| 30 | 4.94 | 1.19 | 42.7 | 57.2 | 55.2 | 9.20 | 7.96 | 17.1 | 3.40 | No |
| Mean | 9.95 | 2.09 | 70.7 | 69.3 | 61.5 | 18.7 | 13.5 | 32.2 | 3.60 | Yes 10 |
| S,D. | 8.29 | 3.79 | 60.3 | 60.7 | 38.4 | 14.1 | 4.38 | 15.2 | 1.08 | No 20 |
| Median | 7.34 | 1.36 | 49.7 | 50.6 | 51.5 | 13.6 | 12.9 | 28.9 | 3.70 |  |
| 1Q-3Q | 5.69 - 10.9 | 0.74 - 1.84 | 35.7 - 88.9 | 32.2 - 83.2 | 32.7 - 87.7 | 10.6 – 25.0 | 11.3 - 15.4 | 21.1 - 38.8 | 3.07 - 4.16 |  |

^a^Abbreviations: ECMO, extracorporeal membrane oxygenation; CL, total clearance; CL_CR_, creatinine clearance; CG, glomerular filtration rate (GFR) by Cockcroft-Gault equation; mMDRD, estimated GFR by MDRD formula adjusted for body surface area (BSA); mCE, estimated GFR by CKD-EPI formula adjusted for BSA; V_C_, central volume of distribution; V_P_, peripheral volume of distribution; V_SS_, steady-state volume of distribution (V_SS_ = V_C_ + V_P_); CRRT, continuous renal replacement therapy

# Supplementary Figures


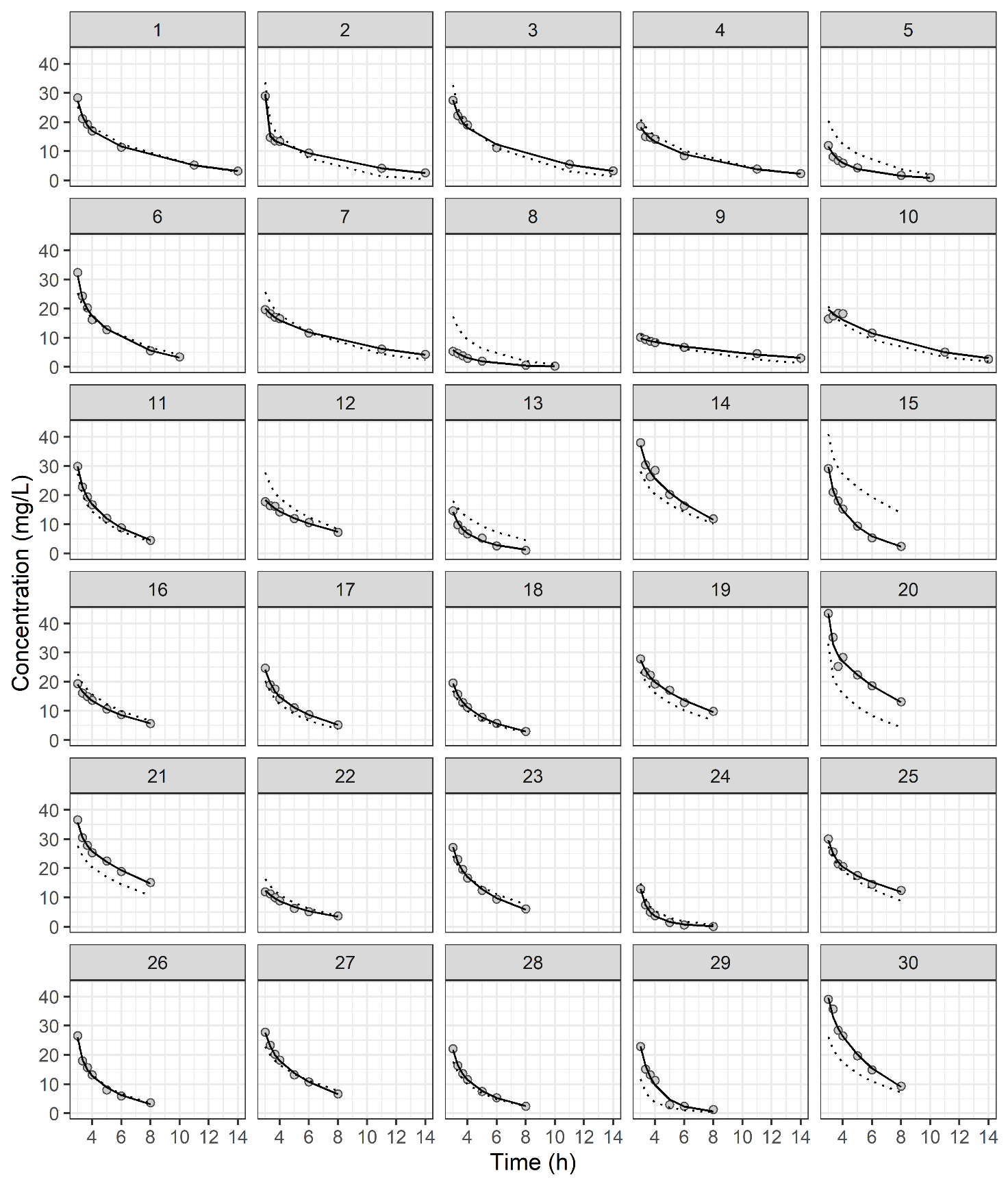


**Supplementary Figure 1.** Individual fit plots: closed circle = observed concentrations; solid line = individual-predicted concentrations; and dotted line = population-predicted concentrations.


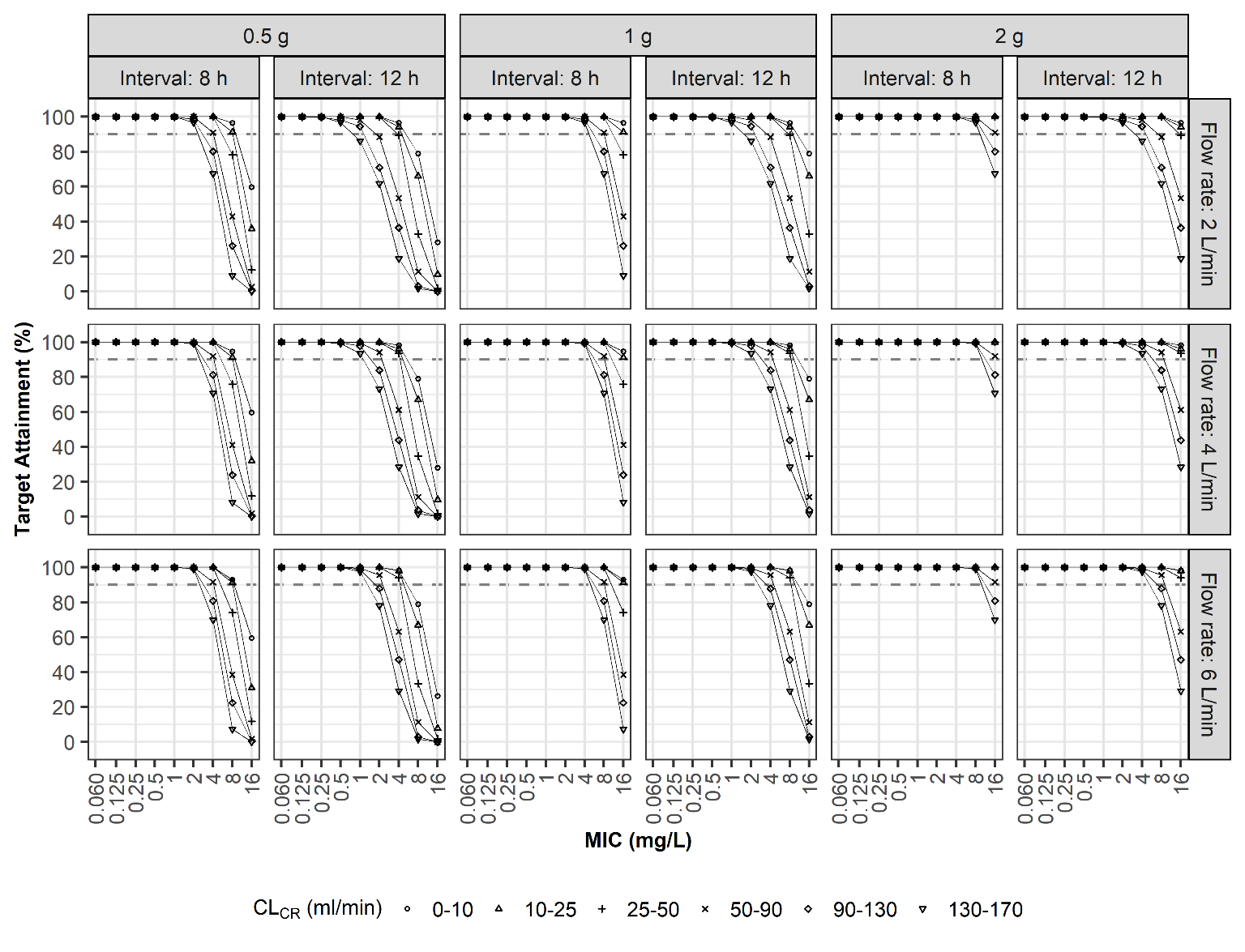


**Supplementary Figure 2.** Probabilities of target attainment (40% *f*T_>MIC_). Monte Carlo simulation results for virtual ECMO patients when using combinations of three doses (0.5, 1, or 2 g), three ECMO flow rates (2, 4, or 6 L/min), two dosing intervals (8 or 12 h), and various MICs and degrees of renal impairment as model inputs. The infusion time is fixed at 0.5 h.
